# Supplementary material for: Pan-Cancer Analysis of Human Kinome Gene Expression and Promoter DNA Methylation Identifies Dark Kinase Biomarkers in Multiple Cancers
Source: Cancers (Basel). 2021 Mar 10;13(6):1189. doi: 10.3390/cancers13061189 (PMC8001681; doi:10.3390/cancers13061189)
Supplement: Supplementary file 1 [file cancers-13-01189-s001.zip › Supplementary_Figures.pdf]

Pan-Cancer Analysis of Human Kinome Gene Expression and Promoter DNA Methylation Identifies Dark Kinase Biomarkers in Multiple Cancers

Siddesh Southekal, Nitish Kumar Mishra * and Chittibabu Guda *

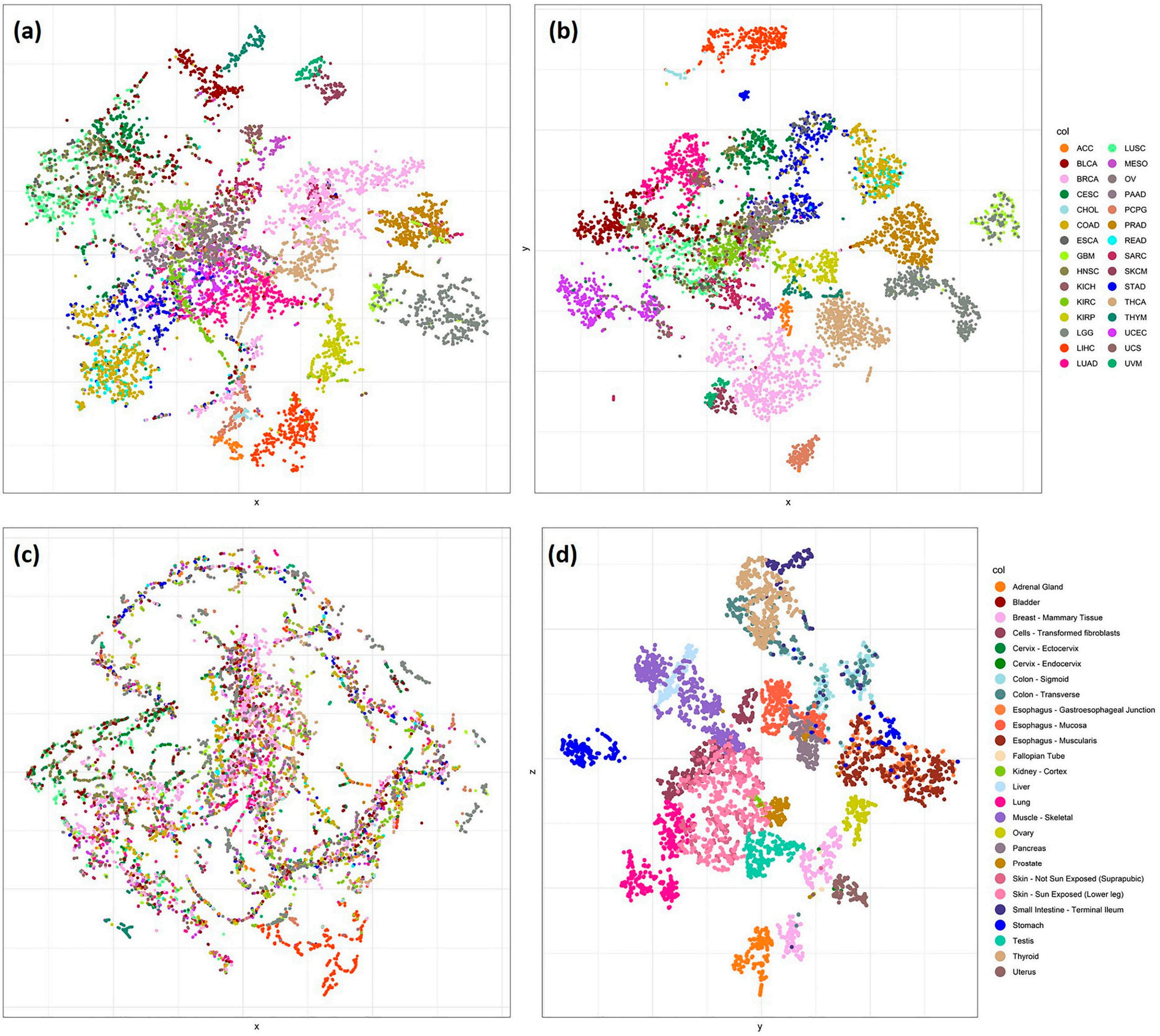

Figure S1: t-SNE plots using multi-omics data (**a**) t-SNE plot using expression of 496 kinase genes in 9372 samples (**b**) t-SNE plot showing organ wise grouping using kinase promoter methylation values of 5774 CpG probes in 8063 samples. (**c**) t-SNE plot showing non grouping of samples using expression pattern of random set of 500 protein coding genes. (**d**) 2D t-SNE plot 491 Kinase genes and 6,199 gene expression data for normal tissues downloaded from GTEx project. Two clusters of Esophagus were observed. One cluster consisted of Esophagus – Mucosa and the other consisted of mix of Esophagus Gastroesophageal Junction and Esophagus Muscularis. Two clusters of Skin were observed – One consisting of mix of Sun exposed (Lower Leg) and not sun exposed (suprapubic) and other from Skin cells – transformed fibroblasts.

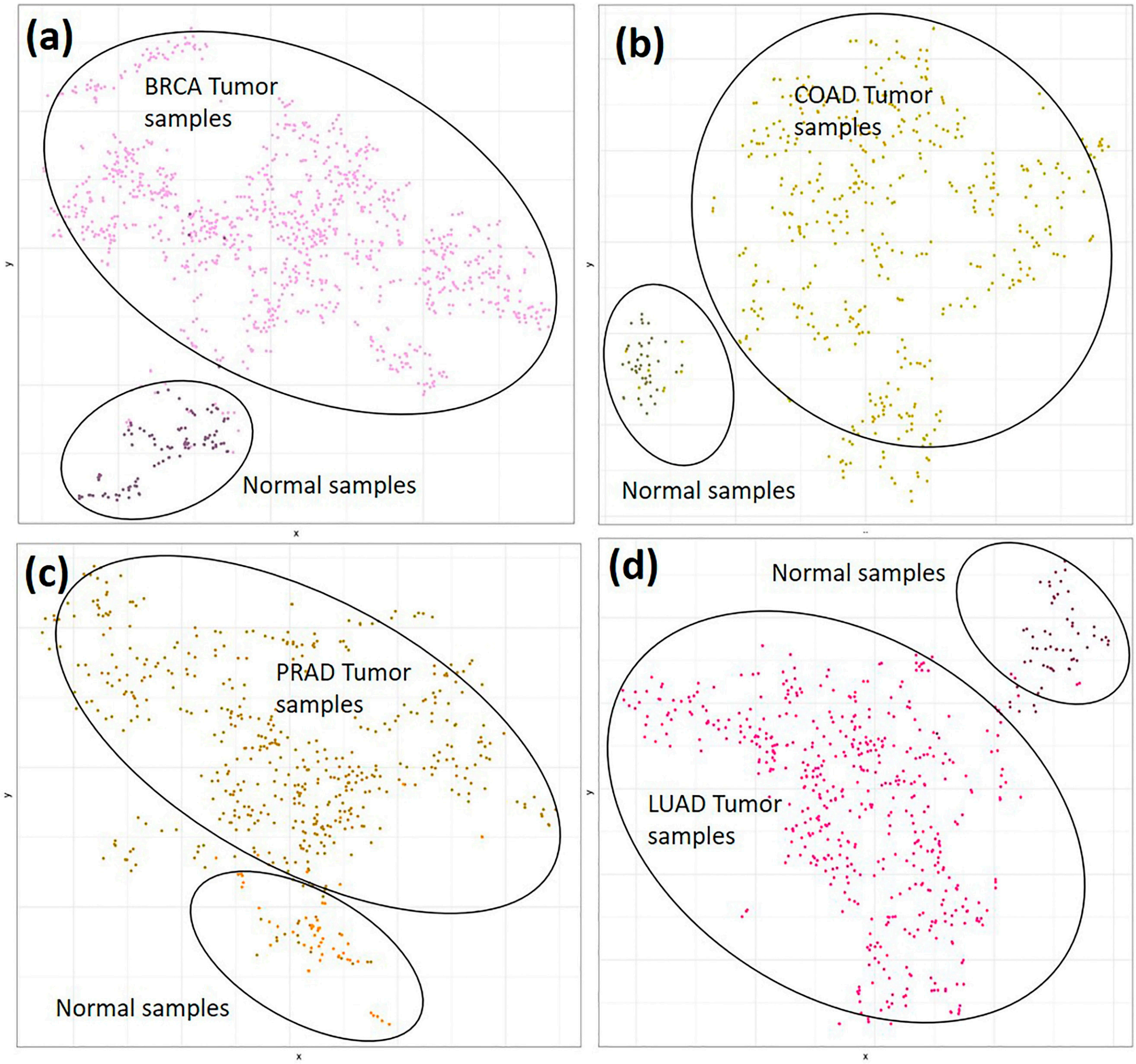

Figure S2: t-SNE plots showing distribution of tumor and normal samples based on expression and methylation data (**a**) breast (**b**) Colon adenocarcinoma (**c**) Prostate adenocarcinoma (**d**) Lung Adenocarcinoma indicating perturbation of kinase gene expression and methylation pattern

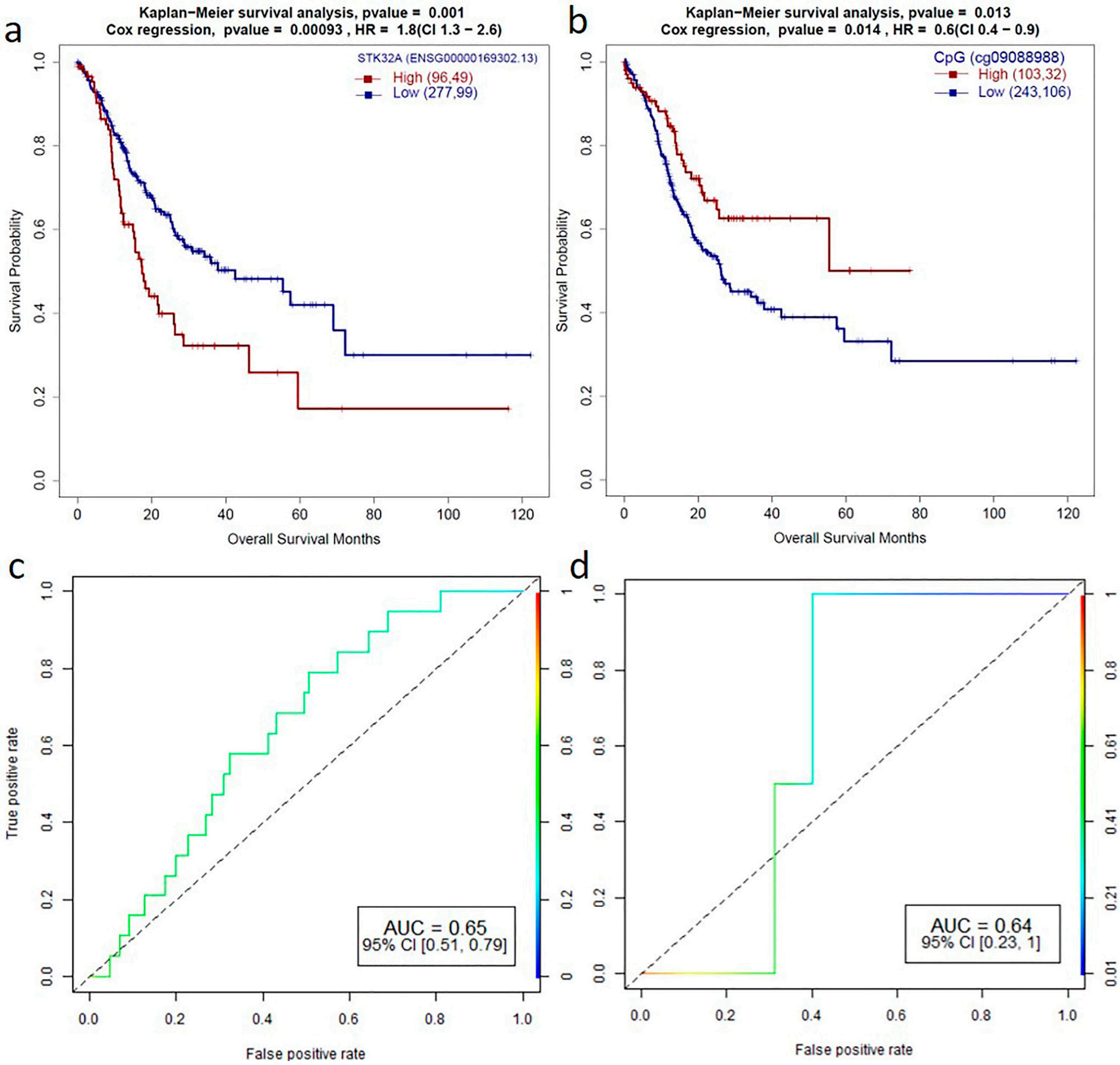

Figure S3: STK32A as prognostic and diagnostic marker in STAD (**a**,**b**) Survival plots of STK32A high Vs low gene expression and promoter DNA methylation sites (cg09088988) which are associated with STAD patient survival with p-value for KM plot (log-rank test) and Cox proportional hazard model (**c**,**d**) ROC plot of gene expression and promoter methylation for the generalized linear model.

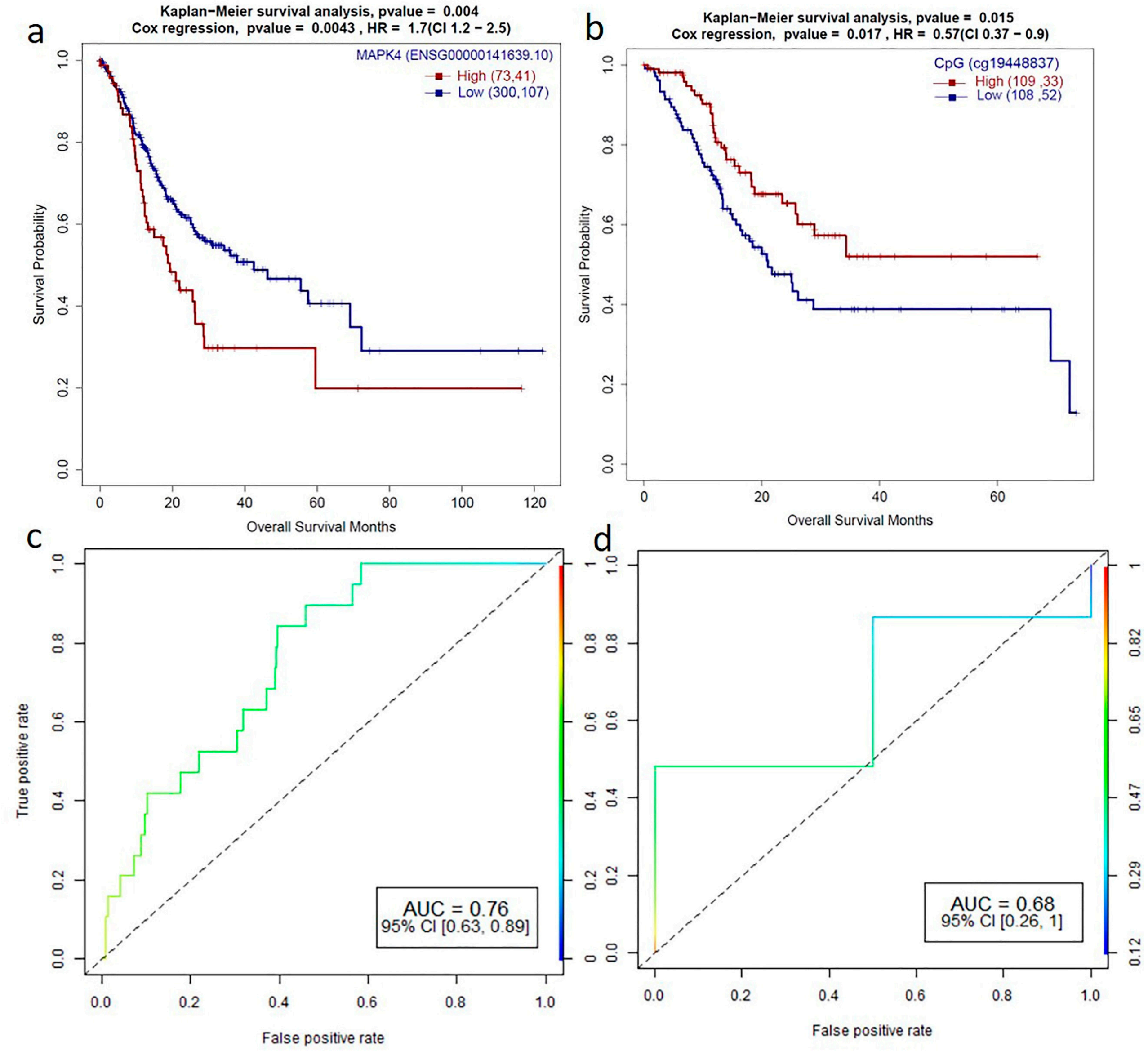

Figure S4: MAPK4 as prognostic and diagnostic marker in STAD (**a**,**b**) Survival plots of MAPK4 high Vs low gene expression and promoter DNA methylation sites (cg19448837) which are associated with STAD patient survival with p-value for KM plot (log-rank test) and Cox proportional hazard model (**c**,**d**) ROC plot of gene expression and promoter methylation for the generalized linear model.
